# Machine Learning Approach Reveals the Assembly of Activated Sludge Microbiome with Different Carbon Sources during Microcosm Startup

**DOI:** 10.3390/microorganisms9071387

**Published:** 2021-06-25

**Authors:** Youngjun Kim, Sangeun Park, Seungdae Oh

**Affiliations:** Department of Civil Engineering, Kyung Hee University, Yongin-si 17104, Korea; joz7000@khu.ac.kr (Y.K.); parksangeun@khu.ac.kr (S.P.)

**Keywords:** machine learning, microcosm, activated sludge, reactor startup, carbon source

## Abstract

Activated sludge (AS) microcosm experiments usually begin with inoculating a bioreactor with an AS mixed culture. During the bioreactor startup, AS communities undergo, to some extent, a distortion in their characteristics (e.g., loss of diversity). This work aimed to provide a predictive understanding of the dynamic changes in the community structure and diversity occurring during aerobic AS microcosm startups. AS microcosms were developed using three frequently used carbon sources: acetate (A), glucose (G), and starch (S), respectively. A mathematical modeling approach quantitatively determined that 1.7–2.4 times the solid retention time (SRT) was minimally required for the microcosm startups, during which substantial divergences in the community biomass and diversity (33–45% reduction in species richness and diversity) were observed. A machine learning modeling application using AS microbiome data could successfully (>95% accuracy) predict the assembly pattern of aerobic AS microcosm communities responsive to each carbon source. A feature importance analysis pinpointed specific taxa that were highly indicative of a microcosm feed source (A, G, or S) and significantly contributed for the ML-based predictive classification. The results of this study have important implications on the interpretation and validity of microcosm experiments using AS.

## 1. Introduction

Activated sludge (AS) processes have commonly been used in full-scale municipal wastewater treatment plants (WWTPs) for the past 100 years. AS processes are considered one of the most successful environmental biotechnologies, serving as a final barrier for preventing undesirable environmental consequences (e.g., eutrophication, harmful algal blooms, dead ecosystems, and waterborne diseases) [1]. Microbial communities are intricately linked to the major ecosystem functions of AS, directly affecting the overall system performance (e.g., removal of bulk organic matter, nitrogen, and phosphorous compounds). There have been many technological research efforts to achieve the development, optimization, and sustainable management of AS processes [2]. Numerous studies are also being performed currently to address ecological questions, because microbial ecology provides the scientific foundation underlying pollutant removal, thereby helping to achieve the system’s practical goals [1].

AS communities with practical and scientific significance have been studied in both laboratory microcosms and field (full-scale WWTPs) experiments [3]. An ecological microcosm is a miniature environment designed for simulating a larger-scale environment of interest. Laboratory microcosms can be easily set up at a low cost, can be strictly controlled under laboratory conditions, and are easily replicated [4]. The precise control and manipulation of variables in replicates help researchers to test hypotheses with statistical power, advancing the mechanistic understanding of AS processes. A common practice in many laboratory microcosm studies begins with the inoculation with raw AS communities taken from those of a full-scale WWTP [5,6]. Through the startup period in the bioreactor, AS communities are eventually acclimated (i.e., changes in the community structure and diversity from those of the raw starting material) to the laboratory conditions. The biological features of acclimated AS are designed to serve as experimental “controls”. The acclimated AS (or those further subcultured in separate replicated settings) are then manipulated by experimental treatments [7,8]. The community features of the “treatment groups” are then compared to those of the experimental controls, allowing a systematic description of the treatment and effect and hypothesis testing with statistical support.

Studies related to the protocols and guidelines of the reactor startups are scarce in the literature, although the startup steps (intentional or not) are essential in many AS microcosms and can affect the long-term performances of the microcosms [9]. For example, AS communities acclimated to laboratory conditions during a startup might have lost their original diversity; however, it is still unclear whether this is significant and, if so, how it can be considered in designing and interpreting experiments. Research questions associated with the startup of microcosm experiments can thus include the following: To what extent do AS communities change in community structures and diversity during a microcosm startup? How can the microcosm startup period be quantitatively determined? While several carbon sources (e.g., glucose) are frequently used for microcosm experiments, what are the impacts of the carbon source on the duration of the startups, the degree of community phenotypes, and the extent of the community structure?

To gain insights into these issues, the present study established three sets of triplicate microcosms developed with three frequently used carbon sources: acetate (A), glucose (G), and starch (S). Since A, G, and S are the small metabolic intermediates of saccharides, unit sugars (monosaccharides), and sugar polymers (polysaccharides), we chose them as carbon sources, because they are frequently used for microcosm experiments and well-represent carbon sources of varying levels of structural complexities among the chemically defined substrates. This study carried out a mathematical modeling approach to quantitatively define the time-dependent dynamic changes in the community phenotypes during the startup period. Time-series 16S rRNA gene sequence data were obtained from the microcosm reactors, followed by machine learning (ML) modeling using the microbiome data. ML-based analyses were employed to advance the current understanding for the effects of carbon sources on the community structure, diversity, and microcosm composition. Overall, the results of this study have implications for the experimental design and interpretation of microcosm studies using AS.

## 2. Materials and Methods

### 2.1. Establishment of AS Microcosms

A bioreactor feed was composed of the following chemicals in one liter: NH_4_Cl (0.6 g); K_2_HPO_4_ (0.34 g); KH_2_PO_4_ (0.6 g); CaCl_2_ (0.05 g); FeSO_4_·7H_2_O (0.09 g); MgSO_4_·7H_2_O (0.27 g); and 10 mL of a 100× trace mineral solution consisting of ZnSO_4_·7H_2_O (0.35 mg), MnSO_4_·H_2_O (0.21 mg), H_3_BO_3_ (2.1 mg), COCl_2_·2H_2_O (1.4 mg), CuCl_2_·2H_2_O (0.07 mg), NiSO_4_·6H_2_O (0.14 mg), and Na_2_MoO_4_·2H_2_O (0.21 mg). The feed also contained a carbon source (3.45 g/L of sodium acetate, 1.83 g/L of glucose, or 1.5 g/L of starch), each of which was designed to provide the same level of organic loading rate [10]. A total of nine reactors were established by an inoculation with an AS sample taken from a local full-scale sequencing batch reactor (SBR) process. Three sets of triplicate reactors were developed with three different carbon source feeds, respectively: acetate (A), glucose (G), and starch (S). The reactors were maintained in sequencing-batch mode (e.g., filling, reaction, and decanting) with a cycle duration of 3.5 days. The reactors were aerated with 3 to 4 mg/L of dissolved oxygen during a reaction period. The reactors were operated with 10.5 days of solid retention time (SRT) and 0.2kg COD/m^3^·d of organic loading rate (OLR), comparable to those of full-scale SBR processes [2].

### 2.2. Mathematical Model for Describing Dynamic Functional Acclimation of AS

The phenotypes of the AS communities were analyzed for 77 days by measuring mixed liquor volatile suspended solids (VSS) and COD removal rates. VSS and COD were measured following the standard methods [11]. These two functions are frequently used indicators of biomass growth and heterotrophic activities [7]. The dynamics of a reactor phenotype (VSS concentration in this study) during a startup period of a microcosm experiment was described using the mathematical model previously developed based on an analogy of a mechanical spring and damper system [12]. A functional feature of a model system could be described as a function of time with the parameters in the model equation. The model was chosen because it could describe any dynamic functional response of a biological system over time (e.g., from system startup to steady state) and provide two quantitative values of the dynamic response: a new functional feature of interest that stabilized after reaching a quasi-steady state and the time period taken to reach the quasi-steady state (Time_ss_). A VSS concentration was used as the functional feature of an AS microcosm over time in this study, resulting in VSS concentrations at a steady state (VSS_SS_). The exact ordinary differential equation and the script written in R that were used in this study were described in reference [12]. The method used is detailed in the Supplementary Method.

### 2.3. 16S rRNA Gene Sequencing and Analysis

Three mixed culture samples were taken from the inoculum AS cultures (designated as I). Triplicate mixed culture samples were sampled at days 28 and 77 from three sets of bioreactors fed by A, G, and S, respectively. Genomic DNA was extracted using the MoBio PowerSoil^®^ DNA isolation kit (MOBIO, Carlsbad, CA, USA). A 16S rRNA gene was PCR-amplified with a universal bacterial primer set that targeted the V3–V5 region (305F–805R). The PCR gene amplicon products were sequenced using Illumina’s MiSeq sequencing platform by Macrogen Inc. (Seoul, Korea). Raw sequence data generated from the sequencer were processed using MOTHUR (v.1.41.0), following the MiSeq SOP pipeline [13,14,15]. In brief, the sequence data were filtered with the parameters: maximum length of homopolymer = 8, maxambig = 0, minimum length = 200, and all other parameters at default settings. The resulting sequences were chimera-checked using chimera.vsearch and taxonomically analyzed using classify.seqs. Chimeric sequences and those taxonomically associated with eukaryotes, chloroplasts, archaea, and mitochondria and those unknown were excluded from further analysis. The remaining sequences, after the exclusions, were grouped into operational taxonomic units using the 97% nucleotide identity cutoff. Alpha-diversity indices were evaluated using rarefaction.single, with a rarefied number of sequences (29,000 sequences per sample). Statistical testing for examining the differential features in the community phenotypes and structure was performed with the Mann–Whitney *U* test using R software.

### 2.4. ML Modeling

Four ML models: logistic regression (LR), support vector machine with linear kernel (SVC Linear), support vector machine with radial basis function kernel (SVC RBF), and random forest (RF) were used for ML modeling with a supervised learning algorithm, as described previously [16], using Scikit-Learn 0.23.2 [17]. The training and test datasets were randomly subsampled from the original family composition dataset generated from the MOTHUR analysis. The subsampling for each dataset was performed 10 times with a size of 500 counts. The stratified split resulted in a total of 210 subdatasets from I (*n* = 30), A (*n* = 60), G (*n* = 60), and S (*n* = 60) using train_test_split From each group of the subdatasets, the training subdatasets (80% of the randomly drawn subdatasets) was used to train each model with a five-fold cross-validation approach with StratifiedKFold, determining the optimum hyperparameters using a random search with RandomizedSearchCV. The prediction performance of each ML model, with the hyperparameters optimized from the five-fold cross-validation, was assessed 100 times with the test (hold-out) dataset (20% of the randomly drawn datasets) using two indices: area under the receiver operating characteristic curve (AUC) and accuracy. The accuracy was determined by dividing the number of correct classifications by the total number of classifications. The receiver operating characteristic curve (ROC) was constructed based on the true positive rate and the false positive rate. The AUC value was the area under the ROC, representing the model’s prediction performance. To enhance the interpretability of the ML modeling prediction, a feature importance value (e.g., usefulness/contribution for predicting a target classification) was estimated for the linear model (LR) using the one-versus-rest approach [18]. For the nonlinear model (RF), SHapley Additive exPlanations (SHAP) [19] was used to assess a feature ranking (e.g., an alternative index for a feature’s contribution for a predictive classification). The mean absolute SHAP impact value was estimated using the python implementation (https://github.com/slundberg/shap, accessed on 3 May 2021).

### 2.5. Nucleotide Sequence Accession Number

The 16S rRNA gene sequence datasets used in this study were deposited in GenBank under the following accession numbers: I_0_1_ (SRS2340223), I_0_2_ (SRS2340241), I_0_3_ (SRS2340242), A_28_1_ (SRS2340238), A_28_2_ (SRS2340243), A_28_3_ (SRS2340193), G_28_1_ (SRS2340219), G_28_1_ (SRS2340221), G_28_1_ (SRS2340217), S_28_1_ (SRS2340190), S_28_1_ (SRS2340187), S_28_1_ (SRS2340206), A_77_1_ (SRS2340236), A_77_2_ (SRS2340194), A_77_3_ (SRS2340191), G_77_1_ (SRS2340222), G_77_2_ (SRS2340214), G_77_3_ (SRS2340224), S_77_1_ (SRS2340188), S_77_2_ (SRS2340185), and S_77_3_ (SRS2340208).

## 3. Results and Discussion

### 3.1. Quantifying Functional Dynamics of AS Microcosms during Startups

**Figure 1:**
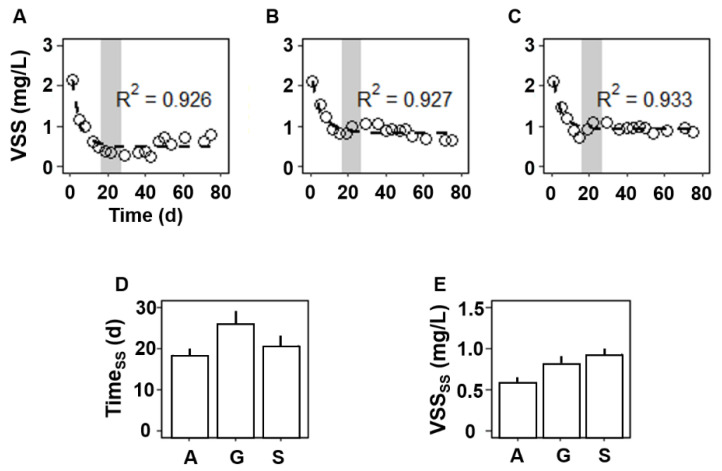
Dynamic functional acclimation during reactor startups. VSS concentrations (open circles) in the A (**A**), G (**B**), and S (**C**) reactors are shown over time. Dotted lines indicate the model predictions. The time periods (Time_ss_) taken to reach the steady state of VSS (**D**) and the VSS (VSS_SS_) levels at the steady state (**E**) were estimated using the model predictions. The range of the Time_ss_ measured with the three carbon sources are shown with shaded areas in (**A**–**C**).

Three sets of laboratory microcosms inoculated from one identical AS culture were maintained over 77 days by feeding A, G, and S, respectively. The COD removals and VSS levels were measured to monitor the microcosms’ phenotypes, because they are practical indicators directly associated with the system performance (e.g., organic matter removal) and an important operational parameter (e.g., biomass growth) in full-scale processes. The COD removals by the three sets of bioreactors were 90–95% over the entire duration (Figure S1) and were not significantly different among the A, G, and S bioreactors. In contrast, the VSS levels of the three bioreactors changed dynamically over time (Figure 1). The initial VSS concentration (2.1 mg/L at day 0) gradually decreased for about a month and then began to level off. The VSS concentrations did not further change significantly after the startup period. The time-course VSS data over the entire operational period (Figure 1) were fitted to the mathematical model [12] with a high goodness of fit (>0.92 of coefficient of determination). The VSS_SS_ was 0.55 ± 0.01, 0.83 ± 0.05, and 0.91 ± 0.03 g/L for A, G, and S, respectively, and the Time_ss_ was 17.7 ± 1.2, 25.0 ± 3.0, and 19.4 ± 2.0 days for A, G, and S, respectively. The modeling approach could quantitatively define the quasi-steady state of a functional feature of interest (i.e., the VSS concentration in this study) after a full-functional acclimation and the minimal time (TSS) taken for reaching the quasi-steady state.

The modeling approach quantitatively determined the degree of community functional change occurring during the microcosm startups. The initial VSS concentration (2.1 g/L) rapidly decreased during the laboratory startups and reached the steady state of 0.55–0.91 gVSS/L. Since the reactor influents contained no microbial inputs, the VSS leaving the reactor (0.18–0.30 gVSS for 3.5 days) should equal that yielded within the reactor at the steady state. The VSS yield was thus 0.32–0.58 gVSS_synthesized_/gCOD_consumed_, considering the stable COD removal rates observed (0.53–0.57 gCOD consumed for 3.5 days). The biomass yield (0.3–0.6 gVSS/gBOD) of the microcosm bioreactors with three carbon sources were comparable to 0.4–0.6 gVSS/gBOD of aerobic heterotrophs in full-scale AS WWTPs [2].

The model predictions could also quantitatively address the minimal period of the microcosm startups, suggesting 18–25 days for the acclimation periods. The time periods for a microcosm startup are often expressed with a unit of SRT. SRT, also known as the mean cell residence time, is the average residence period of microorganisms staying in a system. SRT is an important design and operating parameter for AS processes, because those growing slower than SRT are expelled from the reactor (i.e., those with doubling times longer than the system’s SRT are washed out with the reactor effluents). The startup periods were equivalent to 1.7, 2.4, and 1.8 times the SRT for A, G, and S, respectively, considering the 10.5 days of SRT established in this study. Many previous studies empirically observed 1.5–5 times the SRT as typical acclimation periods for reaching a quasi-steady state in functions associated with the removal of specific substrates (e.g., xenobiotics), nitrogen (e.g., ammonia and nitrite) metabolism, and oxygen uptake [5,6,20], as well as those measured in this study (biomass growth and organic matter removal).

Microbial community acclimation is associated with the dynamic functional changes during startups. The concept of microbial community acclimation is clear, but the quantitative definition remains elusive. Many factors (e.g., type of function to be measured, type of inoculum, and given environmental conditions) can affect the degree and rate of acclimation, which makes quantification challenging. There is no standardized definition/method available for quantitatively determining the acclimation period and the new acclimated functional state. A general rule of thumb for process engineers is that microbial community acclimation in AS microcosms occurs in about three complete reactor turnovers (i.e., about three the times SRT) [3,21], despite variations with distinct operational conditions in each microcosm setting. Little literature about quantification methods for the acclimation period is available. A few studies suggested univariate and multivariate assessment approaches using the biochemical, physical, and morphological characteristics of AS [22,23]. These approaches suggest 20–40 days of minimal periods for microcosm startups (one to two times the SRT in the settings), comparable to those (1.7–2.4 the SRT) measured in our study. Reporting the biological performances of AS microcosms with no clear evidence that the microcosm is at a steady state may hinder the accurate interpretation of the microcosm results. Therefore, the use of a modeling approach, as applied in the previous and present studies, would be highly desirable to ensure whether a bioreactor is at a steady state when reporting a functional feature. Such practices would help not only to draw sound conclusions from the system performance at the given microcosm setting but also enable more accurate comparisons (e.g., meta-analyses) across different AS microcosm studies.

### 3.2. Shifts in Community Structure and Diversity during Laboratory Startups

**Figure 2:**
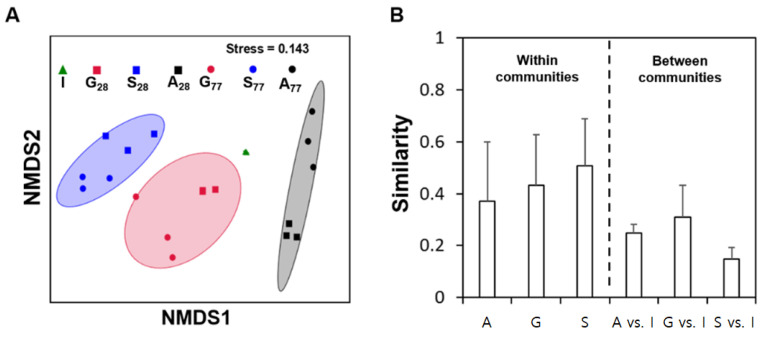
Shifts in the community structure over time. A nonmetric multidimensional scaling plot (**A**) was constructed based on the Bray-Curtis distance metric using the OTU composition data. Six communities developed with a carbon source were grouped within a 68% confidence ellipse. Each of the three groups developed with a carbon source (A, G, or S) differed in community structures from I (*p* < 0.05 by the PERMANOVA test). Community similarities within and between communities (**B**). The community similarities were evaluated within the communities (A, G, and S, respectively) and between the communities (A vs. I, G vs. I, and S vs. I, respectively). The error bars indicate one standard deviation from the mean.

**Figure 3:**
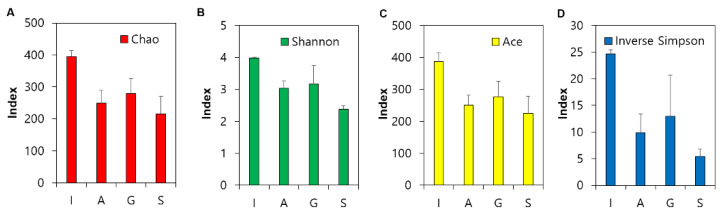
Alpha-diversity indices of the communities: Chao (**A**), Shannon (**B**), Ace (**C**), and Inverse Simpson (**D**).

Although previous studies have documented phenotype changes during AS microcosm startups [22,23], the time-dependent shifts in the community structure, diversity, and composition during startups remain to be further investigated. 16S rRNA gene-based community profiling was thus carried out for the I (at day 0) and AS samples of A, G, and S taken at day 28 (right after the acclimation period) and day 77, respectively. A nonmetric dimensional scaling plot was constructed to illustrate the alterations in the community structure (Figure 2A). First, each group of communities (A, G, and S, respectively) with an identical carbon source clustered closely (i.e., six communities of A, G, and S, respectively, located within their own ellipse) and were clearly distinguished (*p* < 0.05 by the PERMANOVA test) from the I communities, respectively. Figure 2B shows the low community similarity distances (A vs. I, G vs. I, and S vs. I, respectively) between the microcosm and the I communities. Second, although there were detectable divergences between the communities sampled at day 28 (*n* = 3) and at day 77 (*n* = 3) within each group (A, G, or S) (Figure 2A), the degree of the dissimilarity did not reach a typical threshold of statistical significance (*p* > 0.05). Alpha-diversity indices were estimated to assess the shifts in community diversity (Figure 3) after the startups. The Chao1 index values (a proxy of species richness) significantly decreased from the I (395) to A, G, and S communities (215–249) after the startups. The Shannon values were also significantly reduced from I (4.0) in the communities after the startups (2.4–3.0). Other alpha-diversity indices such as Ace (an alternative of Chao1) and Simpson (that of Shannon) showed similar results.

Figure S2 shows the intra-community similarities among the replicates of A, G, and S, respectively, over time. Although the replicate communities were maintained under identical conditions, the community similarities among the replicates decreased in general. These results were consistent with the occurrence of random variations in the community structure among the bioreactor replicates originating from the same inoculum [4]. The decreasing intracommunity similarities even among the replicates may be associated with stochastic (random) variations in the community structure. The community divergence among the replicates increased, but the increasing rates of divergence were differential among the replicates with different carbon sources. While stochastic forces, as well as deterministic ones, contribute to shaping the assembly of microbial communities, the results suggested future experiments on determining the effects of carbon sources on the degree of stochastic forces that drive shifts in the community assembly of AS.

### 3.3. Carbon Source Effects on Community Composition during Microcosm Startups

**Figure 4:**
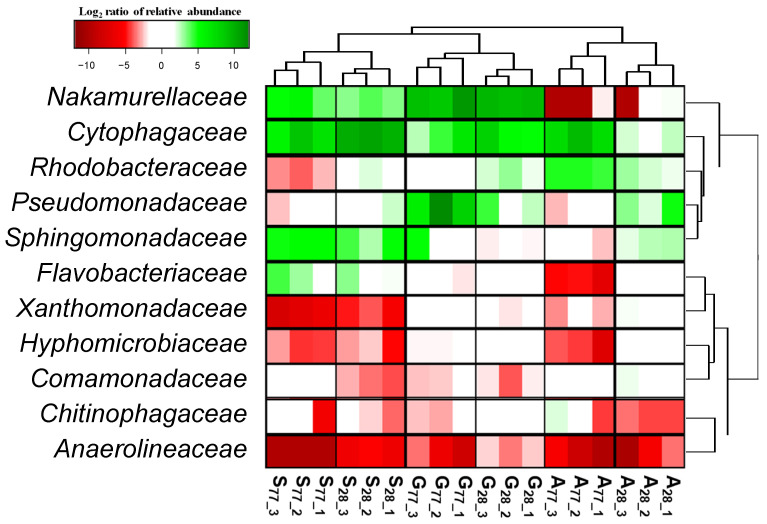
Major families of AS microcosm communities. Eleven major families (>2% of the total, on average) are shown. The Log_2_-transformed fold change (each community over the average of I) is shown by color. Negative/positive values of the Log_2_-transformed fold changes indicate decreased/increased taxa in relative abundance in each community compared to the I communities.

A taxonomic analysis showed that the I communities were dominated by *Bacteroidetes* (33%), followed by *Proteobacteria* (31%), *Acidobacteria* (6%), *Actinobacteria* (5%), *Chloroflexi* (5%), *Planctomycetes* (3%), and *Verrucomicrobia* (1%) (Figure S3). The relative abundance of *Acidobacteria* in I was generally reduced in all microcosm communities (0.3–1.9%). Many *Acidobacteria* are less culturable and/or grow slowly in laboratory conditions [24,25], consistent with selective decreases in their microcosms, as observed in this study. Other major phyla of I were also predominant (>1% on average) in all microcosm communities. The taxonomic analysis further identified 11 major families (Figure 4) that showed differential abundance across the A, G, and S communities. The upper dendrogram of Figure 4 differentiates a group of families overrepresented in I (left) from those selectively enriched (right) in the microcosm communities. Notably, *Anaerolineaceae* and *Hyphomicrobiaceae* were significantly decreased (*p* < 0.05) in the microcosm communities. *Nakamurellaceae* and *Cytophagaceae* were selectively enriched (*p* < 0.05) in both the G and S communities. *Rhodobacteraceae* and *Sphingomonadaceae* particularly dominated (*p* < 0.05) A and S, respectively, compared to I.

**Figure 5:**
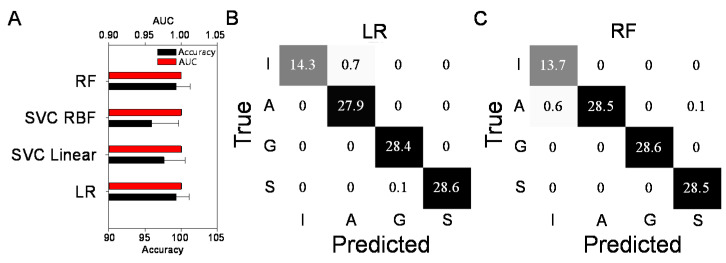
ML modeling prediction performances with the four ML models (**A**) and confusion matrices for the LR (**B**) and RF (**C**) models. The error bars indicate one standard deviation from the mean. The colors in the matrix denote the percentages (presented out of 100).

A ML modeling application using the major family composition data (as shown in Figure 4) was carried out to assess how accurately it could predict the microcosm characteristics (i.e., a feeding carbon source). Four ML models, such as LR, SVC Linear, SVC RBF, and RF, were chosen, since they are widely used models and differ in algorithm complexities (linear vs. nonlinear). Each model was trained with the training dataset to optimize the hyperparameters; after which, the predictive performances with the optimized hyperparameters using the test (held out) datasets were quantified using both the AUC and accuracy indices (Figure 5A). The four models displayed good prediction performances based on the AUC (0.98–0.99) and accuracy (96–99%). Figure 5B,C shows the detailed predictive performances of the two models (LR and RF) with the higher predicting power. The ML-based predictions largely agreed with the actual predictions, mostly positioned on the diagonal boxes (true positives that ML classifications correspond to actual classifications).

**Figure 6:**
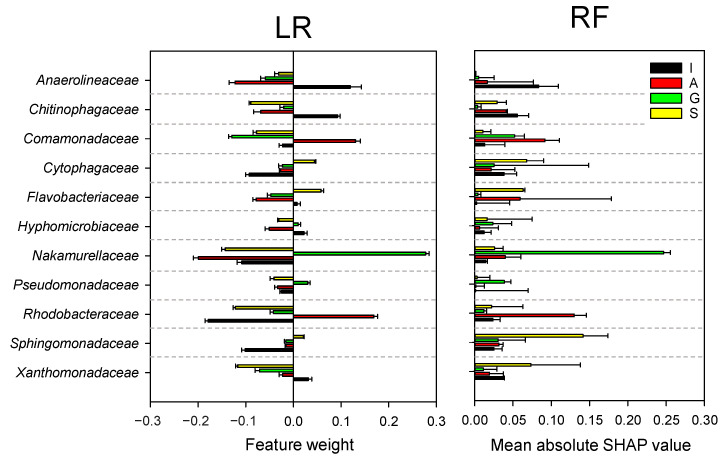
Feature weights and mean absolute SHAP impact values of the major families.

ML modeling was then used to quantify an importance value of each feature (e.g., important family indicators for a feeding source) that contributed to making a predictive classification decision. The feature importance analysis was designed to enhance the interpretability of the ML modeling prediction approach. The analysis was performed using the one-versus-rest approach often used for multicategory classification, which applies a binary classifier for a class against the rest of the other classes. The analysis was performed with the two models with high prediction power, respectively (Figure 6). A family with a positive feature weight value implied strong contribution/usefulness of the family for the predictive classification with the LR model. The analysis identified a list of families strongly associated with each carbon source (*Anaerolineaceae* for I; *Rhodobacteraceae* for A; *Nakamurellaceae* for G; and *Flavobacteriaceae*, *Cytophagaceae*, and *Sphingomonadaceae* for S). The feature importance analysis was also performed with the nonlinear ML model RF, which provided the mean absolute SHAP value of a family for each classification. The SHAP value estimates (Figure 6) suggested the particular significance of *Anaerolineaceae*, *Rhodobacteraceae*, *Nakamurellaceae*, and *Sphingomonadaceae*, for I, A, G, and S, respectively. The RF-based feature importance results were highly consistent with those from the LR model (Figure 6) and conventional statistical testing based on the differential relative abundances (Figure 4).

*Anaerolineaceae* are often found in both natural (e.g., rice paddy soil and marine sediment) and engineered (e.g., activated sludge and anaerobic digester) environments. They can utilize carbohydrates and proteins as electron donors (i.e., chemoheterotrophs) and can optimally grow under both mesophilic and thermophilic conditions [26]. They are strict anaerobes, which likely resulted in their underrepresentation in the microcosm setting of this study, in which 3 to 4 mg/L of DO was strictly maintained. Many AS microcosm experiments are conducted in a single chamber where a redox condition is strictly controlled, as in this study (aerobic). Microcosm experiments with different redox conditions (e.g., adding anoxic/anaerobic chambers in addition to the aerobic one, as in this study) can be considered if organisms with different growth characteristics (e.g., *Anaerolineaceae*) are to be maintained in microcosms. *Hyphomicrobiaceae* are an alphaproteobacterial family group that is phylogenetically diverse (e.g., encompassing 18 known genera) and metabolically versatile [27]. Many of the family members are aerobic/facultative and oligocarbophilic. The oligocarbophilic organisms preferably grow at low levels of carbon and are unable to thrive in rich carbonaceous media. They are frequently found in cellulose-degrading communities, suggesting they have the capability to metabolize complex natural organic matters (e.g., lignocellulose) [28]. Cellulose accounts for about half of organic matter in municipal wastewaters. The underrepresentation of *Hyphomicrobiaceae* in the microcosm communities was likely because the abundance of complex carbohydrates in real waste streams was missing in the microcosm feeds. Many AS microcosm studies are performed with chemically defined single-carbon sources (e.g., glucose and acetate, as in this study) or complex sources (peptone and yeast extract). Depending on the purpose of the experiment, microcosms may be designed to retain those organisms (e.g., *Hyphomicrobiaceae*), preferably utilizing natural complex organic matters, which was not performed in this study and, thus, is the subject of future studies. Those settings can be established by providing broad substrates (a carbon mixture including lignocellulose, more resembling those of real wastewaters).

*Nakamurellaceae* and *Cytophagaceae* were commonly overrepresented in the G and S communities. It was expected that the G and S communities might commonly select some taxa that have competitive fitness for metabolizing a glucose unit, because starches are composed of many glucose units that are joined with a glycosidic bond. Of note was their differential abundance in the A communities, where only *Nakamurellaceae* were significantly underrepresented. A previous study reported some members of *Nakamurellaceae* (*N*. *panacisegetis*) as unable to metabolize acetate as a carbon and energy source [29], implying that they may be less competitive for multiplication in the acetate-enriched condition. *Rhodobacteraceae* are an alphaproteobacterial group widely distributed in marine environments, participating in global biogeochemical cycles (e.g., sulfur and carbon). They are aerobic/facultative anaerobic, characterized by their highly diverse ecological niches (comprising approximately 170 genera) [30]. A stable isotope probing analysis showed that *Rhodobacteraceae* are one of the major acetate-assimilating bacterial groups in AS, in agreement with its selection in the A communities, as observed in this study. The enrichment of *Sphingomonadaceae* in S communities was noticeable. A breakdown of glycosidic linkage is the first and rate-limiting catabolic step for cells utilizing starch as a carbon and energy source. The first metabolic step is carried out by a specific enzyme (e.g., glycoside hydrolase) that transforms complex carbohydrates into simpler forms (e.g., monosaccharides), enabling the transportation of simpler substrates across bacterial membranes. Some members of *Sphingomonadaceae* are characterized by their genetic potential associated with glycoside hydrolase genes [31]; their enzymatic activities in the degrading glycosidic bonds were experimentally confirmed by biochemical assays [32]. Overall, the carbon source used for developing microcosm experiments had a profound impact on shaping the community structure and composition.

### 3.4. Implication on Microcosm Studies Using AS

AS processes are model ecosystems for fundamental microbiological research in ecology, in addition to their practical significance in human societies. Gaining ecological insights from full-scale WWTPs remains challenging because of many confounding factors simultaneously acting at different rates and at varying spatiotemporal scales. Laboratory microcosms provide many exclusive advantages for research; however, a major criticism of microcosms is to what extent the microcosm features (e.g., community diversity) represent those in full-scale WWTPs. This study revealed the reduction of species richness (37–45% by Chao) and diversity (33–40% by Shannon) in microcosm communities (Figure 3). The loss of biodiversity and the alteration in the community structure (Figure 2 and Figure 3) were pronounced, particularly during the startup period, which was well-defined by the predictive model (Figure 1). The three carbon sources frequently used in microcosm settings resulted in a similar duration of the microcosm startup and similar extent of the biodiversity loss and divergence in the community structure. With different carbon sources in microcosm startups, some degree of divergence in the community structure and composition was inevitable.

Wastewater influents contain large amounts of microorganisms that are constantly loaded into the AS process in full-scale WWTPs. These microbial inputs that are hardly simulated in microcosm experiments might contribute, at least in part, to the reduced biodiversity observed in microcosm studies. Nevertheless, these incoming microbial cells (e.g., fecal coliforms) are typically inactive in AS processes (e.g., subject to natural decay out of their human/animal hosts) and not significant contributors to major AS ecosystem functions (contaminant removals). In addition, although AS communities may have many members, not all organisms contribute equally to ecosystem functioning. A keystone species hypothesis suggests that key taxa exist in a community where they play a disproportionately large role in the function and stability of the ecosystem [33]. Other community members largely rely on the keystone taxa, without which the functions of the ecosystem change significantly [8]. Recent studies have shown select core members that are abundant and that highly frequently exist across a variety of full-scale AS processes [34,35,36]. These studies report less than a dozen core species, some of which may presumably serve as keystone organisms driving important ecosystem functions. The relevant question to be asked is whether those core taxa can be retained during microcosm startups. It should be noted that most of the taxa (e.g., *Comanonadaceae*, *Campylobacteraceae*, *Chitinophagaceae*, *Flavobacteriaceae*, *Pseudomonadaceae*, *Rhodocyclaceae*, and *Xanthomonadaceae*) previously defined as core organisms were indeed found in the microcosm communities developed with the three carbon sources, respectively. Rare taxa that are less abundant and are transiently occurring members may also multiply under specific environmental stimuli. For example, rare members are often related to metabolizing small amounts of micropollutants (e.g., pharmaceuticals and industrial chemicals) present in wastewaters [37,38]. Rare members with a low abundance may contribute less to major ecosystem functions [39], despite the debates on the exact roles and importance of these rare members. Accordingly, the core community concept puts a high priority on future experimental efforts for characterizing AS functions, with a particular focus on the core members. Therefore, microcosm studies using AS likely may have relevance and validity for testing ecological/technical concepts if the laboratory microcosms are found to retain core AS members and the purposes of the experiments are not to particularly target specific rare members.

## 4. Conclusions

- A mathematical model revealed 1.7–2.4 times the SRT as the minimal duration for microcosm startups using AS.
- The species richness and diversity indices were reduced by 37–45% and 33–40%, respectively, in the AS microcosm communities.
- The ML modeling application using microbiome data showed high performances (>95% of accuracy) for predicting the assembly patterns of microcosm communities shaped upon feeding carbon sources.
- Despite the inevitable reduction in community diversity, AS microcosm communities might retain many of the core AS members often found in full-scale WWTPs.

## Data Availability

Not applicable.

## Supplementary Materials

The following are available online at https://www.mdpi.com/article/10.3390/microorganisms9071387/s1, Figure S1: Time course COD removal rates, Figure S2: Intra-community similarity among replicates in microcosms over time, Figure S3: Major phyla of microbial communities.
