# Supplementary material for: Machine Learning Approach Reveals the Assembly of Activated Sludge Microbiome with Different Carbon Sources during Microcosm Startup
Source: Microorganisms. 2021 Jun 25;9(7):1387. doi: 10.3390/microorganisms9071387 (PMC8304691; doi:10.3390/microorganisms9071387)
Supplement: Supplementary file 1 [file microorganisms-09-01387-s001.zip › microorganisms-1241829-supplementary.pdf]

SUPPLEMENTARY MATERIAL

Machine learning approach reveals the assembly of activated sludge microbiome with different carbon sources during microcosm startup

Youngjun Kim, Sangeun Park, and Seungdae Oh*

Department of Civil Engineering, Kyung Hee University, Yongin-si, Gyeonggi-do, Republic of Korea

* Author for correspondence

Department of Civil Engineering, Kyung Hee University, Yongin-si, Gyeonggi-do, Republic of Korea

E-mail: soh@khu.ac.kr

Tel: +82 (31) 201 3664

SUPPLEMENTARY METHODS

The full description of the theory, equation derivation, and usage of the mathematical model is described in [1]. The previous study developed the original ordinary differential equation based on the analogy of a mechanical spring and damper system with the application of Newton's second law of motion:

$$m\frac{d^2y}{dt^2} + \lambda\frac{dy}{dt} + ky = kxH(t)$$

where m is the mass, y is the displacement, t is the time, λ is the damping constant, k is the spring constant, x is the final displacement, and H is a unit Heaviside step function. $\omega = (k/m)^{1/2}$ is the natural frequency and $\zeta = \lambda/2(km)^{1/2}$ is the damping factor. The model equation was fitted to the experimental data to estimate the parameters. The fitting was performed using the R script reported previously [1]. The modeling performance of how well the predicted data could approximate the actual data was assessed based on the coefficients of determination (R^2). The model was designed to approximate a dynamic resilient biological response, which was adopted to simulate a dynamic acclimation phenotype in this study. The model fitting with times series VSS data using the R script resulted in the two acclimation phenotype parameters: $Time_{ss}$ and VSS_{ss}. $Time_{ss} = 5\zeta/\omega$ is the time period to reach the steady state and $VSS_{ss} = x$ is the VSS concentration at the steady state.

SUPPLEMENTARY FIGURES

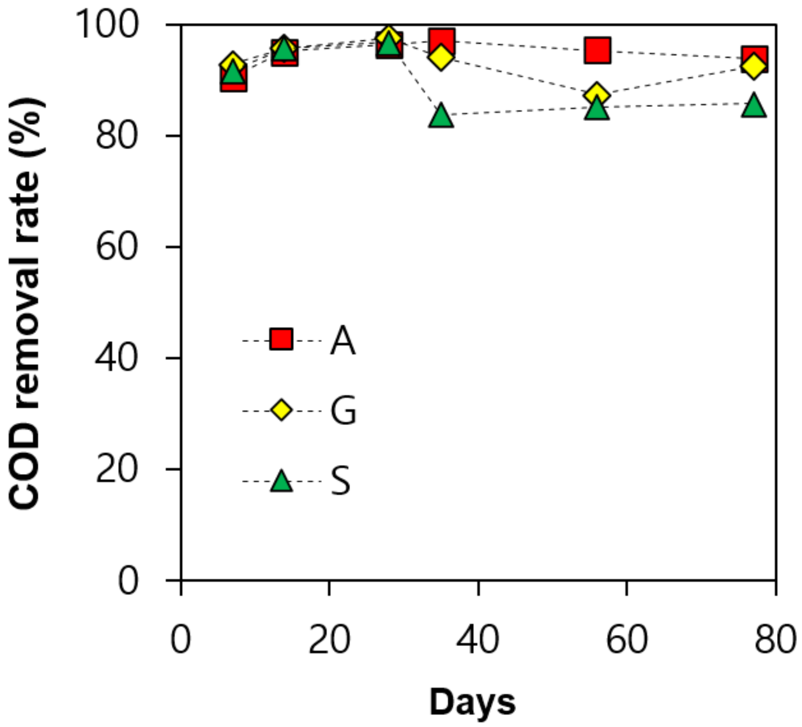

Figure S1. Time course COD removal rates.

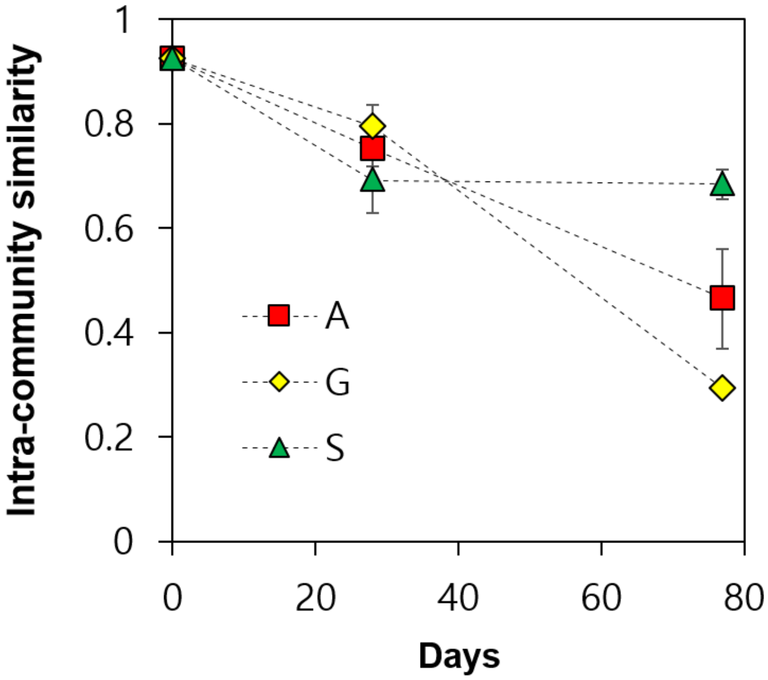

Figure S2. Intra-community similarity among replicates in microcosms over time. The community similarity among triple replicates (A, G, and S, respectively) sampled at day 0, 28, and 77, respectively, was estimated. A strong negative correlation value (Pearson's R < -0.95 with $P < 0.05$) between time and intra-community similarity among replicates was observed for A and G communities, respectively.

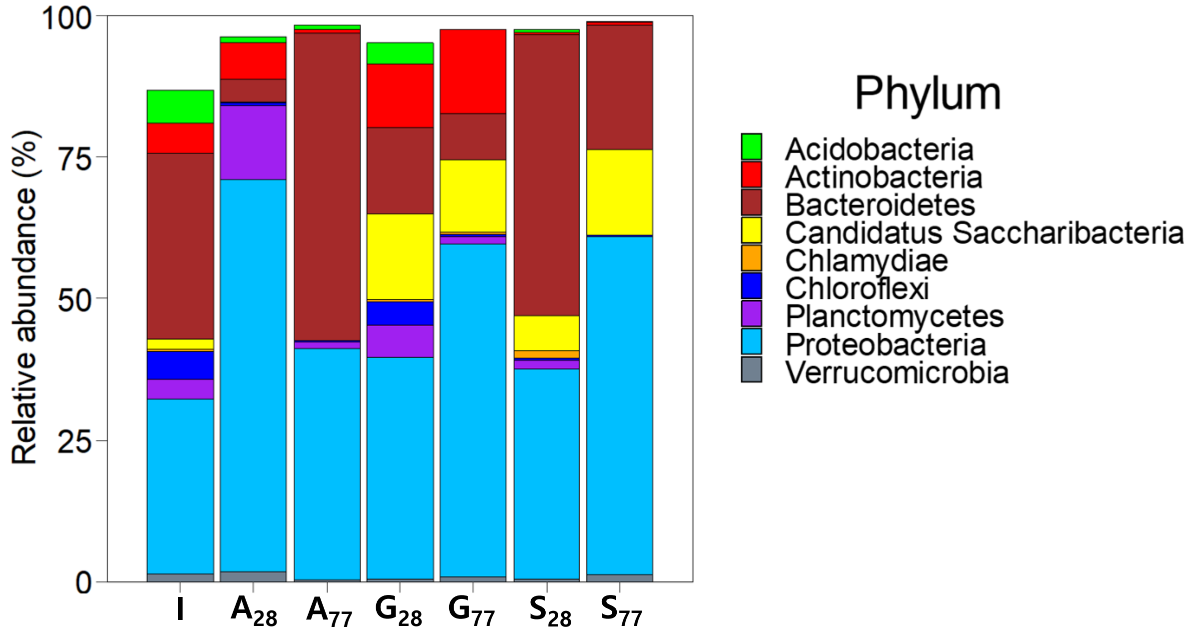

Figure S3. Major phyla of microbial communities. Nine major phyla represent those with > 1% of relative abundance on average.

Reference

1. Todman, L.C.; Fraser, F.C.; Corstanje, R.; Deeks, L.K.; Harris, J.A.; Pawlett, M.; Ritz, K.; Whitmore, A.P. Defining and quantifying the resilience of responses to disturbance: a conceptual and modelling approach from soil science. *Scientific Reports* **2016**, *6*, doi:ARTN 2842610.1038/srep28426.
